# Biomimetic Nanotechnology Vol. 3

**DOI:** 10.3390/biomimetics8010102

**Published:** 2023-03-03

**Authors:** Ille C. Gebeshuber

**Affiliations:** Institute of Applied Physics, TU Wien, 1040 Vienna, Austria; gebeshuber@iap.tuwien.ac.at; Tel.: +43-1-58801-13483

**Keywords:** bacterial capsular gene expression, biomimetic nanotechnology, cardiovascular disease, fungi-mediated nanoparticle synthesis, green synthesis, metallic nanoparticles, nano-herbformulation, origami, quartz crystal microbalance (QCM), Siddha medicine

## Abstract

Biomimetic nanotechnology pertains to the fundamental elements of living systems and the translation of their properties into human applications. The underlying functionalities of biological materials, structures and processes are primarily rooted in the nanoscale domain, serving as a source of inspiration for materials science, medicine, physics, sensor technologies, smart materials science and other interdisciplinary fields. The Biomimetics Special Issues Biomimetic Nanotechnology Vols. 1–3 feature a collection of research and review articles contributed by experts in the field, delving into significant realms of biomimetic nanotechnology. This publication, Vol. 3, comprises four research articles and one review article, which offer valuable insights and inspiration for innovative approaches inspired by Nature’s living systems. The spectrum of the articles is wide and deep and ranges from genetics, traditional medicine, origami, fungi and quartz to green synthesis of nanoparticles.

## 1. Biomimetic Nanotechnology

Biomimetic nanotechnology draws from a wide range of scientific disciplines, including biology, chemistry, materials science and engineering, to design and develop structures, materials and processes that mimic related ones found in Nature. Inspired from the remarkable efficiency, functionality and adaptability of biological systems, researchers and developers active in biomimetic nanotechnology seek to replicate these features in artificial meso-, micro- and nanoscale materials, structures and processes, with functionalities based on the nanoscale and extending to larger scales, sometimes in a hierarchical way, with added functionalities on each level of hierarchy [1]. The goal of biomimetic nanotechnology is to create new technologies that are inspired by the natural systems found in living organisms and use their principles to develop more efficient, sustainable and environmentally friendly solutions for a wide range of applications.

Biomimetic nanotechnology deals with structures and properties that are similar to natural materials, such as cell membranes or spider silk, or the development of devices that mimic the functions of biological systems, such as photosynthesis or chemical sensing. Examples of biomimetic nanotechnology include the development of materials and devices that mimic the properties and functions of cell membranes, such as drug delivery systems or biosensors, the creation of synthetic materials that mimic the mechanical properties of spider silk, and the design of nanoscale systems that mimic the processes of photosynthesis. Properties and functions of biological systems, such as enzymes, cells and tissues, are mimicked in technologically relevant materials. One example are artificial enzymes that are used in a wide range of applications, including biotechnology, medicine and environmental science. Another example for biomimetic nanotechnology is the development of self-assembling nanoscale systems. Such systems can be used to create new materials with specific properties and functions, such as highly efficient catalysts and sensors.

Biomimetic nanotechnology addresses the design, synthesis and characterization of nanoscale materials and devices that mimic the properties and functions of biological systems. Important are self-assembling materials, such as liposomes and dendrimers. As is the creation of materials with responsive or adaptive properties, such as shape-memory polymers and hydrogels. Biomimetic nanotechnology has a wide range of potential applications, including medical devices, environmental remediation, energy conversion and the creation of new materials with improved properties and functionalities. Biomimetic nanotechnology is a rapidly growing field that seeks to replicate the properties and functions of biological systems in artificial materials and systems at the nanoscale. This field has the potential to lead to a wide range of innovations in areas such as biotechnology, medicine and environmental science, and to provide new solutions to some of the most pressing problems facing society today.

As guest editor of the Biomimetic Nanotechnology Special Issues Vol. 1–3 of the MDPI journal Biomimetics [2,3,4] I am happy to introduce the manuscripts selected for publication in Vol. 3, provide a red thread through the articles and give an outlook on the future of this exciting field.

The papers featured in this special issue comprise four original research articles and one review article. In the first article, the authors investigate the 4D printing of self-folding and self-assembling structures as a means to better understand the formation of viral capsids, an area of research that has gained considerable importance in the wake of the COVID-19 pandemic [5]. The second article combines traditional Indian Siddha medicine with nanotechnology to explore novel approaches for treating cardiovascular diseases [6]. The third article is a comprehensive review of fungi-mediated synthesis of nanoparticles, including those of common metals such as Silver and Gold, as well as less commonly studied metals such as Tellurium and Selenium [7]. Such green nanotechnology approaches hold great promise for cleaning contaminated soil and water, and for obtaining valuable resources from the environment. The fourth article investigates the effect of ZnO nanoparticles on the capsular gene expression in *Klebsiella pneumoniae*, a bacterium known for its resistance to a wide range of antibiotics and its potential to cause serious infections in hospital patients [8]. Lastly, the final article presents a study on the molecularly designed imprinting methods for sensing Cu(II) ions using a highly sensitive quartz crystal microbalance, an important approach for detecting environmental toxicants in water [9].

## 2. Jungck et al. on Self-Assembly, Self-Folding and Origami

Jungck et al. deal in their research article “Self-Assembly, Self-Folding, and Origami: Comparative Design Principles” [5] with 4D printing related to modeling and better understanding the formation of viral capsids in vivo. The US American researchers from biology and mathematics departments examine Dürer nets of dodecahedra and icosahedra and their corresponding Schlegel diagrams, and use Hamiltonian circuits and Eulerian paths to develop geometric and topological principles of self-organization and thereby establish new ways of understanding biological and biomimetic self-assembly and self-folding.

**Figure 1:**
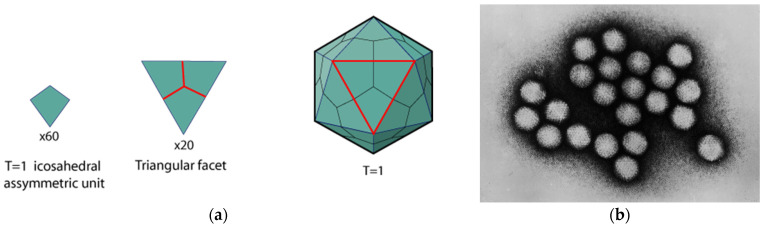
(**a**) Capsid of a model virus composed of 20 triangular facet subunits. Each of the subunits is composed of three icosahedral asymmetric units, resulting in a total of 60 capsid proteins. Source: https://viralzone.expasy.org/1057 (accessed on 28 February 2023), (**b**) An example for a virus with such a capsid is *Adenovirus* which has a diameter of 70 nm–90 nm. Source: Public Domain Image, CDC/Dr. G. William Gary, Jr. Creation Date: 1981. https://commons.wikimedia.org/wiki/File:Adenovirus_transmission_electron_micrograph_B82-0142_lores.jpg (accessed on 28 February 2023).

Self-assembly is important in life. Self-assembly refers to the process in which individual components come together spontaneously to form a more complex structure without external intervention [10]. This concept is crucial for the emergence and maintenance of life, as it is a driving force for the organization and complexity of biological systems, ranging from cells to organisms. Viruses (Figure 1), ribosomes, molecular motors, mitotic spindles, DNA, RNA and protein folding—the assembly of complex structures from elementary building blocks without significant external intervention is ubiquitous in Nature. Such interactions involve non-covalent bonding through ionic interactions, positive-negative polar associations, hydrogen bonding and van der Waals hydrophobic residue interactions.

Furthermore, self-assembly is also involved in the formation and evolution of multicellular organisms. The cooperative behavior of cells during development, tissue maintenance and repair is a result of self-assembly processes that allow cells to coordinate and form functional structures.

Self-assembly is a key principle in biomimetic nanotechnology, allowing for the creation of complex nanoscale structures and devices through the spontaneous organization of individual components. The study of self-assembly in biological systems provides a rich source of inspiration for the development of new self-assembling materials and devices in this field. This process can occur at various scales, ranging from the molecular level to the macroscopic level, and is often used in the fabrication of nanoscale devices and materials.

One example are diatoms: unicellular algae that have a unique ability to create intricate and ornate silica structures known as frustules. These structures are composed of intricate patterns of repetitive multiscale features ranging from 0.3 nm to the centimeter length scale such as pores, ridges, and other features that vary depending on the species of diatom [11]. One of the most fascinating aspects of diatom frustules is their ability to self-assemble. At the nanoscale, diatoms produce silica nanoparticles known as silica nanospheres, which they then assemble into larger structures through a process known as templated self-assembly. This process involves using the nanospheres as a template to guide the growth of larger silica structures, resulting in beautiful highly organized and hierarchical architectures. For example, diatom frustules have been used as templates to create photonic crystals, which have potential applications in sensors, optical communications and other fields [11].

Self-folding refers to the ability of a material to fold itself into a specific shape or form, usually triggered by some external stimulus such as temperature or light. Self-folding materials can be made of a variety of materials, including polymers, hydrogels and metals, and are often used in the development of soft robotics, artificial muscles and micromachines.

A deeper understanding of the underlying principles of self-folding has the potential to impact various fields, including the deployment of emergency shelters in remote locations or even extraterrestrial environments. Barbara Imhof, an Austrian artist and space architect, is an example of an individual who combines biomimetic nanotechnology and artistic concepts in her designs, such as an unfolding lunar station inspired by the ladybird, one of the best-known and most well-loved beetles. The ladybug’s ability to unfold its wings through the use of muscles attached to its body, without external assistance, serves as a source of inspiration for Imhof’s work [12].

In biomimetic nanotechnology, self-assembly is utilized to create nanoscale materials and devices with controlled size, shape and composition. For example, self-assembly can be used to create nanoscale building blocks that can be precisely organized into functional devices, such as sensors, drug delivery systems and electronic components.

4D printing is a technology that combines traditional 3D printing with the ability to create materials that change shape and properties over time in response to external stimuli such as via self-assembly or self-folding. The fourth dimension in 4D printing refers to the dynamic and evolving nature of the materials and structures created using this technology. In 4D printing, special materials are used that are designed to react to changes in their environment, such as temperature, light, or humidity. These materials can be programmed to change shape and properties in specific ways, allowing for the creation of complex and adaptive structures. Thereby, 4D printing represents a new frontier in material science and manufacturing, enabling the creation of dynamic and adaptive structures that can change and evolve over time in response to their environment.

The development of stretch foils used in 3D wrapping and packaging of goods combines traditional 2D foil production techniques with engineered 3D interaction between the individual layers of the material due to their chemical, physical and structural properties. Our current time demands efficient, biobased and recyclable stretch foils [13]. Structuring the foil using natural principles and taking into consideration geometric and mechanical constraints yields new and innovative solutions to the complex challenge of sustainable packaging. Both technologies, 4D printing and 3D wrapping, also rely on the manipulation and folding of materials in order to achieve their desired properties. In the case of 4D printing, this involves the use of materials that can change shape or properties over time, allowing for the creation of complex structures. Similarly, the stretch foil relies on the manipulation of biodegradable materials to achieve its stretchy, protective properties. In both cases, the complex functionalities are a result from simple components. By drawing inspiration from the natural world, researchers and engineers may be able to develop new and innovative solutions to complex challenges, such as sustainable packaging or complex self-assembling structures. The connection between 4D printing and 2D bio-based compostable stretch foil offers a unique opportunity to advance our understanding of self-assembly in nature, and to develop novel materials with enhanced properties for a wide range of applications.

Jungck et al. [14] have asserted that the entropy-driven self-assembly of many biological structures counters traditional narratives that assert biological organization is due to working against the second law of thermodynamics and helps us understand the evolution of many complex biological structures. As Swiegers, Balakrishnan and Huang [15] state: “*Thermodynamic self-assembly therefore has the unique property of being ‘self-correcting’.*”.

One interesting type of organisms for the study of self-folding on the microscale are diatoms. Diatoms are single-celled algae that are known for their intricate and beautiful silica shells, called frustules. These frustules play a crucial role in the survival of diatoms, as they protect the cell from predators and harmful environmental conditions. One of the unique features of some diatom species is their ability to form chains using interlocking devices and hinges. Interlocking devices in diatoms refer to the mechanical locking mechanisms that allow two cells to fit together securely; mechanical hinges connect two objects or surfaces together, allowing them to pivot or rotate relative to each other. Both hinges and interlocking devices in diatoms typically consist of ridges, grooves and other structures that engage with each other [16].

The study by Jungck et al. [5] highlights the significance of structure in biology, biomimetics and engineering, emphasizing the interplay between shape, form and function. Despite the prevalent focus on materials chemistry among scientists, it is imperative to incorporate the concept of structural interactions and structure-based functionalities in the advancement of new technologies. This shift in perspective will provide a more comprehensive understanding of biological systems and related biomimetic applications [17].

## 3. Jayakodi et al. on Nano-Herbformulations in Medicine

The work “Preparation of Novel Nanoformulation to Enhance Efficacy in the Treatment of Cardiovascular Disease” [6] with authors from India, Korea and Ethiopia is an intriguing combination of traditional medicine, modern nanomedicine and state-of-the art analytical techniques. The article describes a new way of delivering Copper to heart cells, as a means to establish a nanoparticle-based drug delivery system utilizing Copper oxide-based nanoherb formulations. The studies are rooted in the ancient Indian Siddha system of medicine and holistic healing. The Copper oxide nanoparticles are synthesized with the help of herbal formulations, resulting in a herbomineral formulation.

Nano drug delivery systems offer the possibility to deliver drugs in a controlled manner, at a specific target site in the body [18]. These systems are designed to improve the efficacy and safety of drugs by enhancing their pharmacokinetic properties. Nano drug delivery systems have several advantages over traditional drug delivery methods. They can increase drug solubility, bioavailability and stability, while reducing toxicity and side effects. Moreover, they can improve drug targeting and reduce drug resistance. Nano drug delivery systems have the potential to revolutionize the field of medicine.

Production of nanoparticles with help of herbal extracts has a long history in nanomedicine (for review see, e.g., [19]). The production of nanoparticles using herbal extracts is an emerging field of research with great potential for various applications, including biomedical and environmental applications. Herbal extracts are rich in bioactive compounds and contain various phytochemicals that can act as reducing and stabilizing agents for the synthesis of nanoparticles.

There are various methods used for the synthesis of nanoparticles using herbal extracts, including green synthesis, microemulsion-based synthesis and solvothermal synthesis. Green synthesis involves the use of herbal extracts as reducing and stabilizing agents for the synthesis of nanoparticles [20]. For example, the extract of *Garcinia mangostana* has been used to synthesize Gold nanoparticles [21]. Microemulsion-based synthesis involves the use of microemulsions as a medium for the synthesis of nanoparticles using herbal extracts [22]. For example, the extract of *Curcuma longa* has been used to synthesize Silver nanoparticles using this method [23]. Solvothermal synthesis involves the use of high temperature and pressure in a solvent-based system to synthesize nanoparticles using herbal extracts [24]. For example, the extract of *Phyllanthus emblica* has been used to synthesize Zinc oxide nanoparticles using this method [24]. The use of herbal extracts in the synthesis of nanoparticles is a promising area of research with great potential for various applications. Amongst further positive effects, the resulting herbal formulations are supposed to be less toxic [25]. The toxicity of nanoparticles prepared using herbal extracts compared to those prepared using standard synthesis methods can vary depending on several factors, such as the composition of the herbal extract, the method used for synthesis and the type of nanoparticle. Further research is needed to fully understand the mechanisms involved in the synthesis of nanoparticles using herbal extracts and to optimize the methods for large-scale production.

In general, nanoparticles prepared using herbal extracts are considered to be less toxic compared to those prepared using chemical synthesis methods. This is because the bioactive compounds present in the herbal extracts can act as stabilizing agents for the nanoparticles, reducing their toxicity. In addition, herbal extracts are typically biocompatible and less likely to cause adverse effects compared to the chemical agents used in standard synthesis methods.

However, the toxicity of nanoparticles prepared using herbal extracts can still depend on various factors, such as the size and shape of the particles, the dose and duration of exposure and the route of administration. As with any new technology, it is important to carefully evaluate the safety and toxicity of nanoparticles prepared using herbal extracts before they can be used in applications such as medical or environmental applications.

It is also important to note that not all herbal extracts are safe for use in the synthesis of nanoparticles. Some extracts may contain toxic compounds that can be harmful when incorporated into nanoparticles. Therefore, it is important to carefully evaluate the safety of the herbal extract before using it for the synthesis of nanoparticles.

Copper has been traditionally used in various cultures as a remedy for various health conditions, including heart disease. However, there is limited scientific evidence to support its effectiveness as a treatment for heart disease.

The study involves the development of a new type of nanoscale formulation for the treatment of cardiovascular disease. The authors used their knowledge of materials science, nanotechnology, biology and drug delivery to design and synthesize a nanomaterial that shall be capable of effectively treating this disease. The material used in the study is a nanoherb formulation with Copper Oxide, the size of the spherical nanomaterial ranges from 20 nm to 50 nm, the best way to functionalize the material to enhance its performance and reduce toxicity identified by the authors is a green chemistry approach of production via herbal extracts. The authors characterized the properties of the nanoscale formulation to ensure that it has the desired size, shape and surface properties. This includes UV-vis spectroscopy studies, XRT and FTIR, scanning electron microscopy combined with EDX techniques as well as gas chromatography mass spectrometry and in vitro studies on cell lines.

The authors propose their novel nanoformulations as a promising approach for the treatment of cardiovascular disease.

## 4. Loshchinina et al. Review Fungi-Mediated Synthesis of Nanoparticles

The second article on biosynthesis in this special issue “Biomimetic Nanotechnology Vol. 3” of the MDPI scientific journal Biomimetics is a review article on the diversity of biogenic nanoparticles obtained by the fungi-mediated synthesis written by researchers from Russia [7].

Fungi-mediated synthesis falls under the category of biogenic synthesis, which is a type of green synthesis that uses living organisms such as fungi, bacteria or yeast to synthesize nanoparticles. In biogenic synthesis, living cultures or various substances derived from organisms are used to reduce metal ions to metallic nanoparticles or to synthesize metal-organic or metal-polymeric nanoparticles.

Biogenic synthesis offers several advantages over other synthesis methods, including the use of easily accessible and renewable resources, the ability to synthesize particles with unique and tunable properties, and the absence of toxic and hazardous chemicals.

The work “Diversity of Biogenic Nanoparticles Obtained by the Fungi-Mediated Synthesis: A Review” is a review article that focuses on the diversity of biogenic nanoparticles that can be obtained through fungi-mediated synthesis.

The authors of this work conducted a comprehensive review of the literature published between 2018 and 2022 to gather information on the various types of biogenic nanoparticles that can be obtained through fungi-mediated synthesis. This includes information on the types of fungi used, the synthesis methods, and the properties and applications of the resulting nanoparticles.

**Figure 2:**
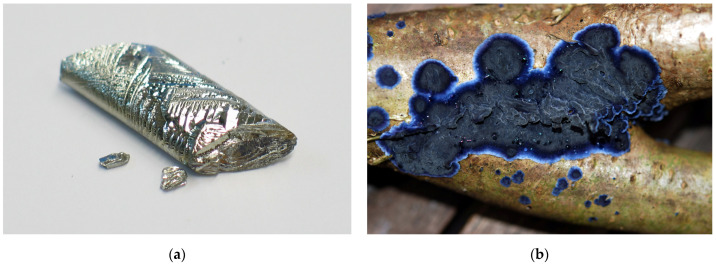
The crust fungus *Phanerodontia chrysosporium* can be used to synthesize needle-like Tellurium nanoparticles: (**a**) Crystal of the chemical element Tellurium (Wikimedia Commons, user Dschwen, source: https://upload.wikimedia.org/wikipedia/commons/8/8b/Tellurium_crystal.jpg (accessed on 28 February 2023)); (**b**) Crust fungi can be quite colorful, such as this example of the corticoid fungus *Terana caerulea*. Image Source: Martin Bemmann, Wikimedia Commons CC BY 3.0.

Exemplarily, one single fungus shall be featured here, and some of its capabilities introduced: *Phanerodontia chrysosporium* (previously *Phanerochaete chrysosporium*) (Figure 2). Needle-like Tellurium nanoparticles of Te^0^ are found in the hyphae of *Phanerodontia chrysosporium* when incubated with TeO_3_^2−^ [26]. Tellurium (Figure 2) is a rare chemical element that was discovered in 1783 by Franz-Joseph Müller von Reichenstein. The name of this metal is derived from the Latin word “tellus” meaning Earth. The main uses of Tellurium are in solar panels and thermoelectric devices. *Phanerodontia chrysosporium* is a fungus that is widely used in biotechnology and for purification of waste waters. The peroxidases and oxidases that it produces degrade compounds in toxic wastes, e.g., in warm compost piles. This is possible because *Phanerodontia chrysosporium* has a high optimum temperature.

Loshchinina and co-workers stress in their review article the dependence of varying synthesis conditions in the same culture on size, shape, stability and biological activity of the produced nanoparticles. Fungi can produce metals, metalloids, metal oxides and sulfides and other compounds as well as composite nanoparticles in large quantities. They do this with the help of enzymes and other biologically active molecules (such as polysaccharides, proteins or peptides) both within their mycelium as well as extracellularly. Especially the extracellular production is of interest because the organisms does not have to be destroyed to obtain the nanoparticles of interest. The authors critically review myconanosynthesis of Silver, Gold, Platinum, Palladium, Copper, Iron, Selenium and Tellurium nanoparticles, and give extensive related references to key publications published between 2018 and 2022.

The authors provide an overview of the current state of the field, discussing the types of nanoparticles that can be obtained, the methods used, and the properties and applications of these nanoparticles.

Plants, animals, fungi and microorganisms can besides the production of nanoparticles also be utilized for bioremediation and phytomining, to clean the soil from certain toxic materials and also to biomine for certain rare, dangerous or expensive materials such as Rare Earth elements, Gold, or Silver for biomimetic resource management [27]. One of the record holders in this aspect is the hyperaccumulator plant *Alyssum murale* can accumulate Ni up to 20,000 mg kg^−1^ dry weight, and a biomass of 10,000 kg/ha can be harvested per year [28].

## 5. Kudaer and Co-Workers on the Influence of Zink on Gene Expression

The article “Effect of Zinc Oxide Nanoparticles on Capsular Gene Expression in *Klebsiella pneumoniae* Isolated from Clinical Samples” [8] by authors from Iraq deals with the impact of Zinc oxide nanoparticles on the gene expression of *Klebsiella pneumoniae*, a type of bacteria that can cause infections in humans. The researchers studied the effects of such nanoparticles on the capsule, the protective layer that some bacteria produce to evade the host’s immune system. Four genes responsible for capsule manufacturing were investigated, three of them were chromosomal, one of them is found in plasmids.

Plasmids and chromosomal genes are two distinct types of genetic material in cells: Plasmids are small, circular pieces of DNA that are separate from the bacterial chromosome. They are found in bacteria and some eukaryotic cells and can carry genes for antibiotic resistance, virulence, or other traits that are beneficial to the cell. Plasmids can replicate independently from the chromosomal DNA and can be easily transferred from one cell to another, which can contribute to the spread of antibiotic resistance and other traits in populations of bacteria. Chromosomal genes, on the other hand, are located on the chromosomes. Chromosomal genes are passed on to daughter cells when a cell divides, and they encode the majority of the genetic information that determines the traits and characteristics of an organism. In eukaryotes, chromosomal genes are housed within the nucleus, while in prokaryotes they are found in the cytoplasm.

The research was done on real-world bacteria and not just laboratory-grown strains: The authors used about 30 *K. pneumoniae* strains isolated from clinical samples collected from different hospitals in Baghdad/Iraq. This research provides new insights into the mechanisms behind the antibacterial effects of Zinc oxide nanoparticles, and how they can be used to combat *K. pneumoniae* infections.

The authors studied the effect of three different concentrations of Zinc oxide nanoparticles on the gene expression of four genes that are responsible for capsule manufacturing in *K. pneumoniae*. New methods in medicine are necessary, because hospital germs and multidrug-resistant bacteria are bacteria found in hospitals and other medical facilities that are resistant to many antibiotics. They pose a major risk to patients, especially those with weakened immune systems or chronic diseases.

The main result of the study is that the transcript levels of the examined genes and thereby the reproduction of *K. pneumoniae* were reduced when the ZnO nanoparticle concentration was increased and thereby new evidence is presented that could suggest a solution to the crucial issue of antibiotic resistance based on effective bacterial antibiotics based on nanoparticles.

## 6. Aydoğan et al. on Using QCM and MIT to Detect Copper Ions

The article “Molecularly Designed Ion-Imprinted Nanoparticles for Real-Time Sensing of Cu(II) Ions Using Quartz Crystal Microbalance” [9] by authors from Turkey introduces the combination of a molecularly designed imprinting method with a quartz crystal microbalance (QCM) for the detection of Cu(II) ions in aqueous solutions.

Molecular imprinting technology (MIT) is a process used to create molecularly imprinted polymers (MIPs), which are artificial receptor molecules designed to selectively bind to a specific target molecule [29]. The process involves synthesizing a polymer in the presence of the target molecule or a similar molecule, known as a template. During the polymerization process, the template molecule is surrounded by monomers, which form a complex with the template. Once the polymerization is complete, the template molecule is removed, leaving behind a cavity or imprint in the polymer that has a complementary shape and chemical functionality to the template molecule. The resulting MIP can selectively bind to the target molecule, even in complex mixtures, due to the specific shape and chemical properties of the imprint. This makes MIPs useful in a variety of applications, such as sensors, separations and drug delivery. Molecular imprinting technology is highly selective, has good stability and low cost.

The QCM method is a high resolution mass sensing technique, basing on a linear connection between mass change and resonance frequency change.

In the study presented in our Special Issue Biomimetic Nanotechnology Vol. III the authors used a combination of MIP and QCM for experimental studies on Cu(II) ions complexed with N-methacryloyl-L-histidine methyl ester (MAH) monomer in two different mole ratios, both computationally and analytically. The results are promising and the authors recommend such sensors for use in water quality monitoring.

## 7. The Future of Biomimetic Nanotechnology

In the Biomimetic Nanotechnology Special Issues Vol. 1–3 of the MDPI journal Biomimetics [2,3,4] various interesting and promising aspects of the field are presented. As we look to the future, there are several areas where biomimetic nanotechnology is likely to play an increasingly important role: nanoscale drug delivery, energy harvesting and storage, sensing and diagnostics and materials science. In terms of nanoscale drug delivery, deeper understanding of the underlying biological aspects will yield biomimetic nanotechnology-based nanoscale drug delivery systems that mimic the behavior of biological systems even better, allowing for targeted, safe and controlled delivery of therapeutic agents to specific cells or tissues in the body, combined with biocompatibility and biodegradability after application. Concerning energy harvesting and storage, biomimetic nanotechnology shall provide nanoscale materials that can mimic the structure and function of biological systems that efficiently convert and store energy (such as the use of sunlight for the production of glucose and oxygen). Especially in sensing and diagnostics, organisms can be great inspiration for novel sensitive engineering approaches: thresholds of senses are single molecules for smelling and single photons for seeing. By mimicking the structure and function of biological receptors and enzymes, nanosensors shall detect low concentrations of molecules of interest, enabling early detection of disease markers and environmental pollutants. In materials science nanoscale structures that mimic the strength and flexibility of biological materials (such as nacre, spider silk or bones) shall enable the development of new lightweight and durable materials for use in a range of applications. One of the ultimate goals in biomimetic nanotechnology shall be the production of materials and devices we people need with local materials, under ambient conditions, with water-based chemistry, that serve as food or fertilizer at the end of their usage.
